# A case report of primary bilateral macronodular adrenal hyperplasia caused by a novel *ARMC5* gene variant

**DOI:** 10.3389/fmed.2026.1921766

**Published:** 2026-09-23

**Authors:** Yu Tang, Dongxue Li, Juan Xia, Yuan Zhong, Zixia Ni, Liwei Tong

**Affiliations:** 1 Department of Endocrinology, Zigong Fourth People’s Hospital, Zigong, Sichuan, China; 2 Department of Endocrinology and Metabolism, The Affiliated Hospital of Southwest Medical University, Luzhou, Sichuan, China; 3 Department of Health Management Center & Institute of Health Management, Sichuan Provincial People’s Hospital, School of Medicine, University of Electronic Science and Technology of China, Chengdu, Sichuan, China; 4 Department of Endocrinology, Chengdu Second People’s Hospital, Chengdu, Sichuan, China

**Keywords:** *ARMC5* gene variant, case report, Cushing syndrome, novel variant, PBMAH

## Abstract

**Introduction:**

**:** Primary bilateral macronodular adrenal hyperplasia (PBMAH), also known as adrenocorticotropic hormone-independent macronodular adrenal hyperplasia, is a rare cause of endogenous Cushing’s syndrome (CS) in clinical practice. The pathogenesis of PBMAH is complex; however, *ARMC5* variants have been increasingly recognized as important pathogenic genetic factors, as reflected by the growing number of related studies.

**Case presentation:**

A 59-year-old man was admitted because of long-standing poor glycemic and blood pressure control. Endocrine evaluation showed suppressed plasma adrenocorticotropic hormone (ACTH) levels, loss of the circadian cortisol rhythm, and failure of cortisol suppression after the low-dose dexamethasone suppression test. Imaging revealed multiple bilateral adrenal nodules. Histopathological examination showed nodular lesions composed predominantly of clear, lipid-rich cells, with a small number of cells with eosinophilic cytoplasm at the periphery of the nodules. Genetic testing identified an *ARMC5* c.2125C > T (p.Q709*) nonsense variant. Based on the clinical manifestations and laboratory findings, the patient was diagnosed with PBMAH. The patient underwent left unilateral adrenalectomy, and outpatient follow-up showed reductions in antidiabetic and antihypertensive medications, with effective improvements in glycemic and blood pressure control.

**Conclusion:**

This case confirms PBMAH caused by a novel nonsense variant in *ARMC5*, thereby expanding the variant spectrum of *ARMC5*. In patients with long-standing poorly controlled blood glucose and blood pressure, hypokalemia, and recurrent infections, related subclinical endocrine abnormalities should be considered, even in the absence of overt Cushingoid features. A diagnosis can be established through integrated endocrine evaluation, imaging assessment, and genetic testing. This case highlights the importance of screening for secondary endocrine causes in patients with hypertension and diabetes, particularly when clinical or biochemical findings suggest cortisol excess, and may inform clinical management and familial genetic counseling.

## 1 Introduction

Primary bilateral macronodular adrenal hyperplasia (PBMAH) is a rare cause of Cushing's syndrome. Patients may present with hyperglycemia, hypertension, hypokalemia, and/or unexplained recurrent infections (1, 2). Approximately 20%–55% of patients with PBMAH harbor variants in the *ARMC5* gene (2). *ARMC5* has been clearly identified as a tumor suppressor gene, and the development of PBMAH is thought to follow the classic “two-hit” hypothesis. The first hit is a germline variant, which is present in all cells of the patient; the second hit is a somatic alteration that usually occurs in the adrenal gland and may manifest as loss of heterozygosity (LOH) or a nonsense variant affecting the second allele (3). Ultimately, biallelic inactivation leads to complete loss of ARMC5 protein function, thereby abolishing its inhibitory effects on adrenocortical cell proliferation and steroidogenesis. This process drives the formation of asymmetric bilateral adrenal macronodules and autonomous cortisol secretion. In this case, whole-exome sequencing identified a novel nonsense variant in *ARMC5*, c.2125C > T (p.Q709*). A review of the available literature and public databases indicated that this variant has not been previously reported. This finding expands the variantal spectrum of *ARMC5* in PBMAH and may provide a useful reference for early screening, clinical diagnosis, and genetic counseling for family members.

## 2 Case presentation

A 59-year-old man was admitted to our hospital with a more than 10-year history of elevated blood glucose and blood pressure and a 3-month history of poor glycemic control. He had been diagnosed with type 2 diabetes mellitus and hypertension more than 10 years earlier and had received long-term oral medication and insulin therapy as an outpatient. Five years earlier, the patient had been admitted to the hospital for poor glycemic control, with a blood glucose level of 20 mmol/L. Evaluation revealed an abnormal circadian rhythm of cortisol secretion, and the 1-mg overnight dexamethasone suppression test showed nonsuppressible cortisol secretion. Abdominal CT (Figure 1) demonstrated irregular enlargement of both adrenal glands with multiple nodules and masses, the largest measuring approximately 2.4 cm × 2.6 cm × 4.3 cm. Some nodules appeared to have coalesced, and the lesions showed homogeneous and marked enhancement after contrast administration. However, the patient did not attach importance to these findings and declined further diagnostic evaluation and treatment. Three months earlier, the patient was readmitted to the hospital for poor glycemic control, with a blood glucose level of 19 mmol/L. Further history taking revealed a complicated medical history. Five years earlier, he had undergone surgical resection for tuberculosis of the tongue. Four years earlier, he had undergone resection of the lingular segment of the left lung for a left pulmonary nodule, and postoperative pathological examination confirmed tuberculoma of the left upper lobe. Postoperatively, he received oral rifampicin and isoniazid as anti-tuberculosis therapy. More than five months earlier, he had been diagnosed with cryptococcal meningitis because of headache and abnormal behavior, and was treated with oral fluconazole tablets as antifungal therapy. On admission, physical examination showed a temperature of 36.6 °C, pulse rate of 65 beats/min, respiratory rate of 18 breaths/min, and blood pressure of 107/78 mmHg. No purple striae, ecchymoses, or acne were observed on the skin or mucosa. The patient had central obesity and moon facies. Cardiopulmonary examination revealed no obvious abnormalities, and the abdomen was mildly distended. Pulsation of the bilateral dorsalis pedis arteries was weakened. Temperature sensation was decreased, whereas pressure and pinprick pain sensations were normal. Bilateral ankle reflexes were absent. Laboratory findings during endocrine and metabolic evaluation (Table 1): Laboratory tests showed hypokalemia, with a serum potassium level of 2.26 mmol/L (reference range, 3.5–5.3 mmol/L). The standing aldosterone-renin test showed an aldosterone level of 65.136 pg/mL, a renin level of 3.857 pg/mL (reference range, 3.8–38.8 pg/mL), and an aldosterone-to-renin ratio of 8.78 (reference value, ≤30). Assessment of the circadian cortisol rhythm showed loss of the normal diurnal variation, with serum cortisol levels of 19.46 μg/dL at 08:00, 18.31 μg/dL at 16:00, and 17.64 μg/dL at 24:00. ACTH levels were suppressed throughout the day: <1.000 pg/mL at 08:00, <1.000 pg/mL at 16:00, and 1.028 pg/mL at 24:00. The dehydroepiandrosterone sulfate level was decreased at 31.61 μg/dL, whereas the 17α-hydroxyprogesterone level was slightly elevated at 2.42 ng/mL. The body fat percentage was 29.6%. Transcutaneous oxygen pressure measurement suggested bilateral lower-limb ischemia. Vibration perception threshold testing showed severe impairment in the left foot and mild-to-moderate impairment at the other tested sites, while the ABI was 1.0. Both the 1-mg overnight and standard low-dose dexamethasone suppression tests showed nonsuppressible cortisol secretion. Abdominal enhanced CT (Figure 2) shows that the bilateral adrenal glands are obviously irregular and beaded, and there are multiple round-like density reduction shadows in them, which is slightly larger than the image on July 25, 2019.

**Figure 1:**
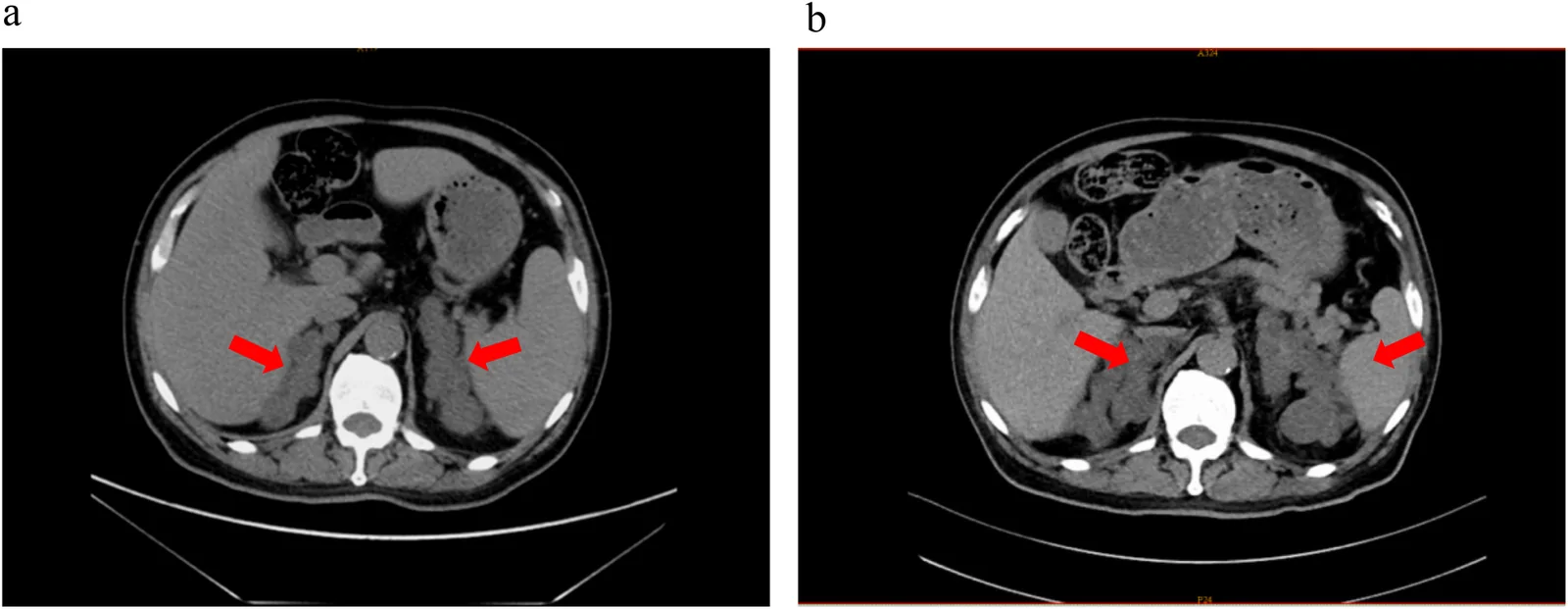
**(a)** Abdominal non-contrast CT performed on January 24, 2023, revealed marked irregular beaded enlargement of the bilateral adrenal glands, accompanied by multiple rounded hypodense lesions. **(b)** Abdominal non-contrast CT on January 11, 2024, revealed multiple well-circumscribed nodular hypodense lesions in both adrenal regions, with the largest lesion measuring approximately 2.5 × 2.2 cm.

**Figure 2:**
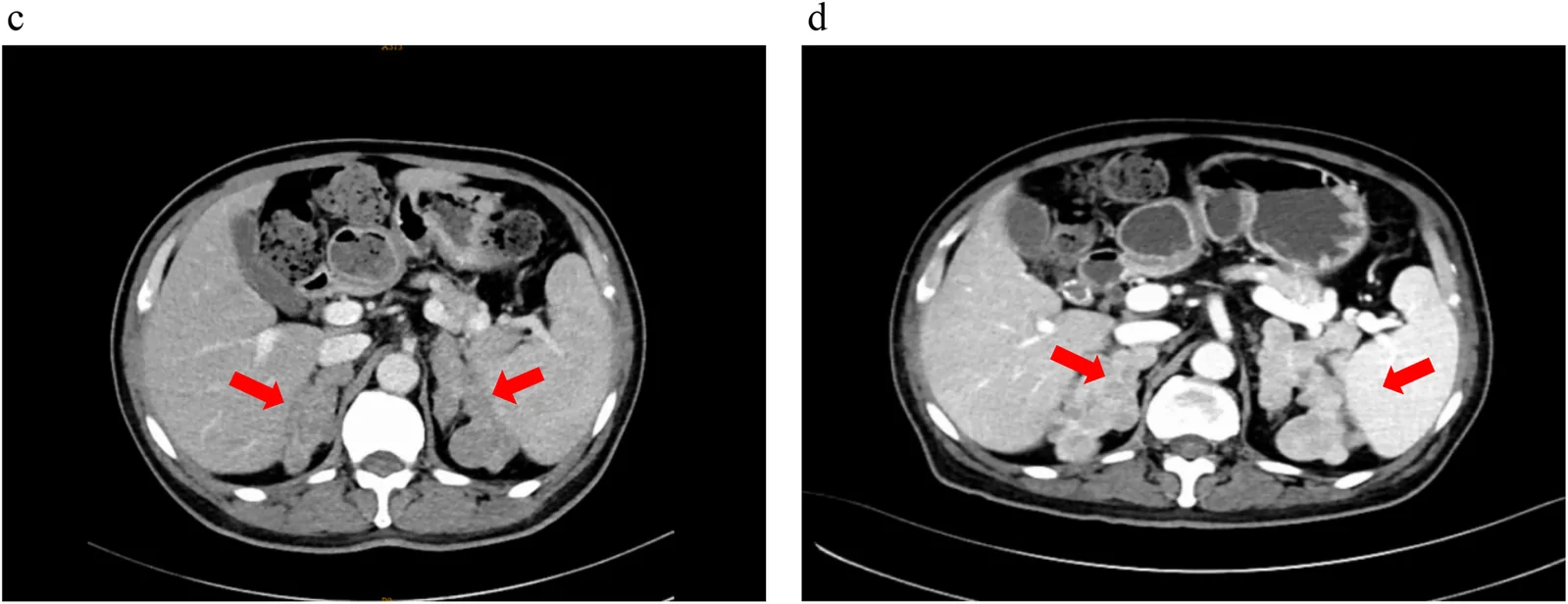
**(c)** Contrast-enhanced abdominal CT image obtained on march 16, 2019, showing irregular nodular thickening and mass-like lesions in both adrenal glands, with inhomogeneous persistent enhancement. **(d)** Contrast-enhanced abdominal CT image obtained on August 1, 2024, showing multiple well-defined, round-to-beaded hypodense lesions in both adrenal glands. The lesions demonstrated marked persistent enhancement on contrast-enhanced images, particularly in the venous phase, with an attenuation of approximately 140 HU.

**Table 1:** Laboratory findings during endocrine and metabolic evaluation.

Category	Parameter	Result	Reference range
Hematological parameters	Leukocyte count	6.41	3.5–9.5 × 10⁹/L
	Platelet count	241	100–350 × 10⁹/L
	Hemoglobin	141	130–175 g/L
	Erythrocyte count	4.87	4.3–5.8 × 10¹²/L
Inflammatory markers	C-reactive protein	5.8	0–6 mg/L
	Procalcitonin	0.052 ↑	0–0.046 ng/mL
Renal function	Estimated glomerular filtration rate	94.62	≥60 mL/min
	Creatinine	76.6	57–97 μmol/L
	Uric acid	194 ↓	208–428 μmol/L
Coagulation marker	D-dimer	0.32	<1.0 mg/L DDU
Liver function	Alanine aminotransferase	34.0	9–50 U/L
	Aspartate aminotransferase	22.0	15–40 U/L
	γ-Glutamyl transferase	96 ↑	10–60 U/L
Glucose metabolism	Fasting blood glucose	8.38 ↑	3.9–6.1 mmol/L
	HbA1c	7.77 ↑	4.0%–6.0%
Lipid metabolism	Triglycerides	3.51 ↑	≤2.3 mmol/L
	Total cholesterol	5.85 ↑	≤5.17 mmol/L
	High-density lipoprotein cholesterol	0.77 ↓	≥1.04 mmol/L
Electrolytes	Serum potassium on admission	2.97 ↓	3.5–5.3 mmol/L
	Serum potassium 2 days after admission without intervention	2.26 ↓	3.5–5.3 mmol/L
	Serum potassium 6 h after potassium replacement therapy	2.97 ↓	3.5–5.3 mmol/L
	Serum potassium 2 days after potassium replacement therapy	3.10 ↓	3.5–5.3 mmol/L
Autoimmune/rheumatologic markers	Anti-streptolysin O	14	0–200 U/mL
	Rheumatoid factor	40.0 ↑	0–20 U/mL
Renin–aldosterone system	Renin, standing position	3.857	3.8–38.8 pg/mL
	Aldosterone, upright position	65.136	pg/mL
	Aldosterone-to-renin ratio, standing position	8.78	≤30
Adrenal androgen/steroid precursor	DHEA-S	31.61 ↓	48.20–362.80 μg/dL
	17*α*-Hydroxyprogesterone	2.42 ↑	0.29–2.06 ng/mL
Catecholamines	Dopamine	41.00 ↑	<30 pg/mL
	Epinephrine	5.18	Supine <111 pg/mL; standing <141 pg/mL
	Norepinephrine	24.30	Supine 70–750 pg/mL; standing 200–1,700 pg/mL
HPA axis assessment	ACTH, 08:00	<1 ↓	7.2–63.4 pg/mL
	ACTH, 16:00	<1 ↓	7.2–63.4 pg/mL
	ACTH, 24:00	1.028 ↓	7.2–63.4 pg/mL
	Serum cortisol, 08:00	19.46	4.3–22.4 μg/dL
	Serum cortisol, 16:00	18.31 ↑	3.09–16.66 μg/dL
	Serum cortisol, 24:00	17.64 ↑	<5 μg/dL
Dexamethasone suppression testing	Serum cortisol after 1-mg overnight DST, 08:00	20.79	See note
	Serum cortisol after low-dose DST, 08:00	19.98	See note
Urinary cortisol	24 h urinary free cortisol	1,042.69 ↑	20–100 μg/24 h
	24 h urinary free cortisol after low-dose DST	1,607.10 ↑	20–100 μg/24 h

ACTH, adrenocorticotropic hormone; ARR, aldosterone-to-renin ratio; CECT, contrast-enhanced computed tomography; DHEA-S, dehydroepiandrosterone sulfate; DST, dexamethasone suppression test; HbA1c, glycated hemoglobin; HPA, hypothalamic–pituitary–adrenal; PCT, procalcitonin; RF, rheumatoid factor.

↑ indicates a value above the reference range; ↓ indicates a value below the reference range. For dexamethasone suppression testing, failure to suppress serum cortisol was interpreted according to commonly used diagnostic criteria; a post-DST serum cortisol level >1.8 μg/dL was considered unsuppressed.

Treatment course: The patient was treated with subcutaneous Humalog Mix 25, 20 U in the morning and 20 U in the evening, in combination with acarbose capsules 50 mg three times daily for glycemic control. For blood pressure control, nifedipine sustained-release tablets 20 mg once daily, sacubitril/valsartan 50 mg once daily, and metoprolol succinate sustained-release tablets 23.75 mg once daily were administered. Potassium chloride sustained-release tablets 1.0 g once daily were given for potassium supplementation. Further evaluation, including urinary catecholamine measurement and a high-dose dexamethasone suppression test, was recommended; however, the patient declined these investigations. After multidisciplinary discussion, the patient underwent three-dimensional laparoscopic left adrenalectomy under general anesthesia on August 14, 2024. Histopathological examination of the left adrenal gland revealed nodular adrenal cortical hyperplasia (Figure 3). At ultra-low magnification (2×), the lesion showed a nodular architecture, with the largest nodule measuring more than 10 mm. At high magnification (40×), the nodules were composed predominantly of clear, lipid-rich cells, with scattered eosinophilic cells at the periphery. The patient subsequently underwent genetic testing. Whole-exome sequencing (WES) identified one phenotype-related variant in *ARMC*5: c.2125C > T (p.Q709*) (Figure 4). This variant was further confirmed by targeted orthogonal validation using Sanger sequencing (Figure 5). On the basis of the clinical presentation, biochemical and hormonal findings, histopathological features, and genetic results, a diagnosis of PBMAH was established. After discharge, the patient completed five outpatient follow-up visits, during which glycemic control and blood pressure improved substantially (Table 2).

**Figure 3:**
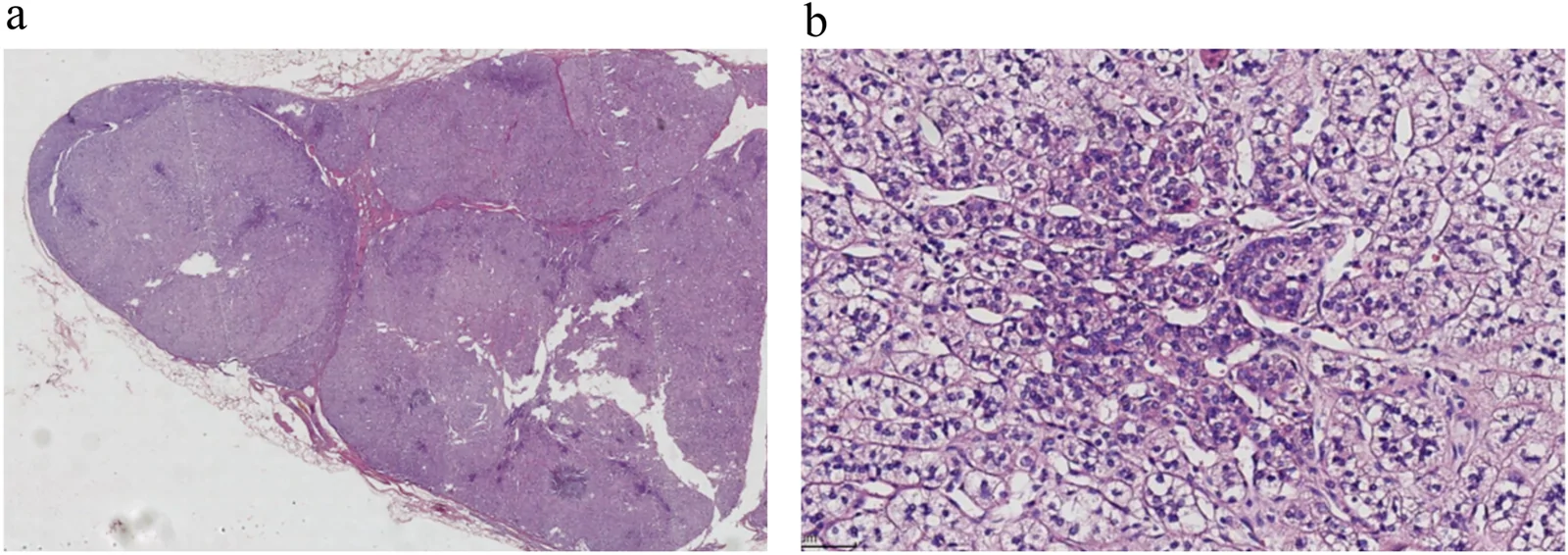
**(a)** Ultra-low-power view (2×) showing nodular lesions, with the largest nodule exceeding 10 mm. **(b)** High-power view (40×) showing nodules composed predominantly of clear, lipid-rich cells, with scattered eosinophilic cells around the nodules.

**Figure 4:**
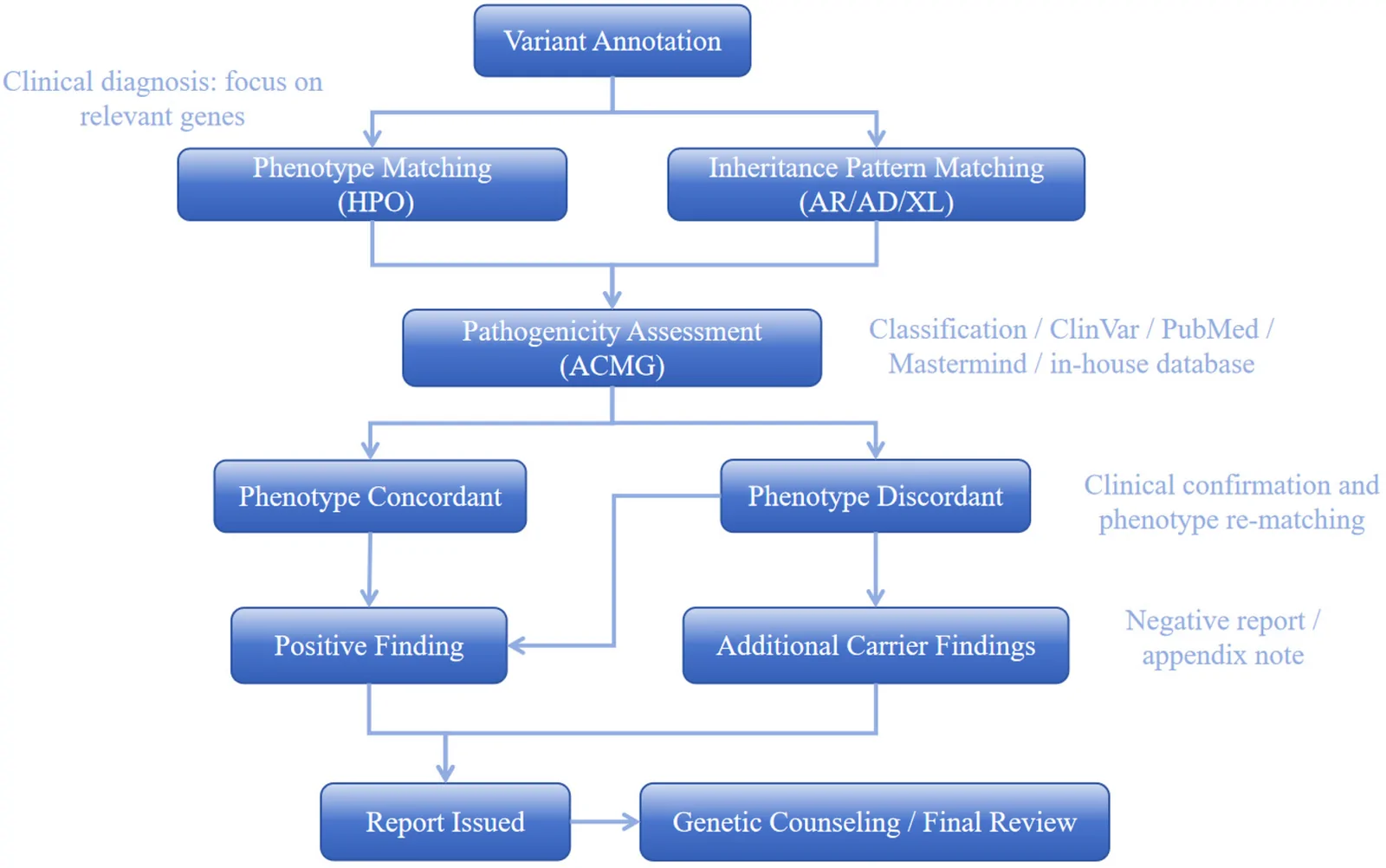
Reference databases: OMIM database, ClinVar database, PubMed database, mastermind database, decipher database, ClinGene database.

**Figure 5:**
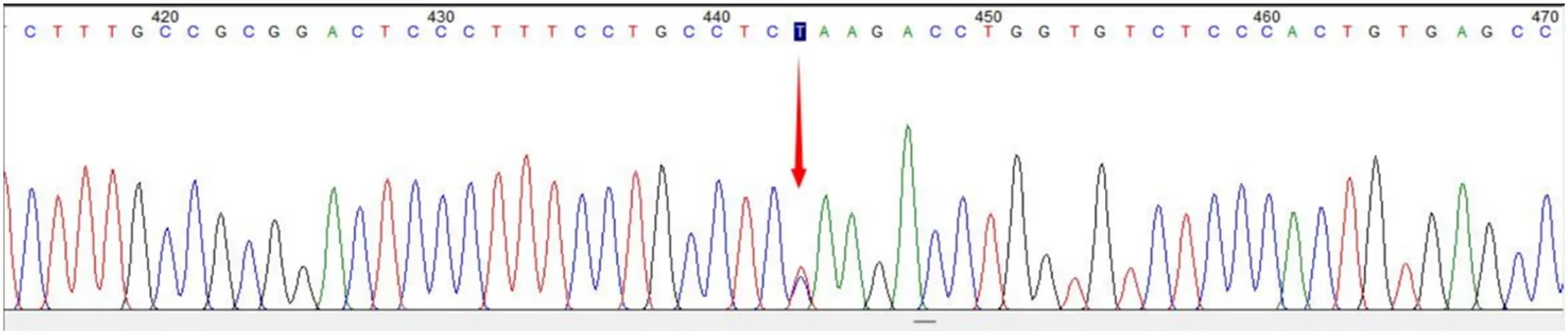
This variant was detected by Zhejiang Shengting Medical Laboratory Co., Ltd. Initial screening by whole-exome sequencing identified a heterozygous *ARMC5* variant, c.2125C > T (p.Q709*), in the patient. This figure shows the Sanger sequencing chromatogram used to validate this variant.

**Table 2:** Summary of follow-up data and medication adjustments.

Postoperative follow-up time Postoperative follow-up index		2024-09-12 Outpatient follow-up	2024-09-24 Outpatient follow-up	2024-10-15 Outpatient follow-up	2024-11-07 Outpatient follow-up	2026-03-30 Outpatient follow-up
Blood pressure fluctuation	**BP(mmHg)**	115/80	113/79	114/76	100/80	120/80
Blood glucose fluctuation	**FPG(mmol/L)**	5.0+	5.0+	6.0-9.0+	8.0-9.0+	5.0+
	**PPG(mmol/L)**	7.0+	7.0+	7.0-8.0+	7.0+	6.0+
Laboratory examination	**ACTH(pg/mL)**	<1.000	\	\	3.877	5.009
	**cortisol(ug/dL)**	6.87	\	\	11.30	8.54
	**renin(pg/mL)**	2.720	\	\	\	46.904
	**aldosterone(pg/mL)**	69.359	\	\	\	69.359
	**ARR**	13.26	\	\	\	1.32
	**17α-OHP(ng/mL)**	2.93	\	\	\	1.35
Treatment		Humalog Mix 25, 15 U before breakfast and 15 U before dinner; previous regimen: Humalog Mix 25, 24 U before breakfast and 24 U before dinner, plus insulin glargine 16 U at bedtime. Acarbose capsules, 50 mg three times daily; atorvastatin calcium, 10 mg once nightly; amlodipine tablets, 5 mg once daily.	Humalog Mix 25, 15 U before breakfast and 15 U before dinner; acarbose capsules, 50 mg three times daily; atorvastatin calcium, 10 mg once nightly; and amlodipine, 5 mg once daily.	Humalog Mix 25, 14 U before breakfast and 14 U before dinner; acarbose capsules, 50 mg three times daily; atorvastatin calcium, 10 mg once nightly; and amlodipine tablets, 2.5/5.0 mg once daily.	Humalog Mix 25, 8 U before breakfast and 8 U before dinner; atorvastatin calcium, 10 mg once nightly; amlodipine, 5.0 mg once daily; acarbose capsules discontinued.	Humalog Mix 25, 8 U before breakfast and 8 U before dinner; atorvastatin calcium, 10 mg once nightly; irbesartan, 150 mg once daily; and amlodipine tablets, 5.0 mg once daily.

## 3 Discussion

PBMAH is a rare adrenocortical disorder characterized by autonomous cortisol hypersecretion, which may lead to endogenous Cushing syndrome (CS). Owing to its insidious clinical manifestations and low prevalence (4), PBMAH is often underdiagnosed in clinical practice, particularly in patients presenting with nonspecific metabolic complications. This report describes a sporadic case of PBMAH presenting with refractory hyperglycemia, hypertension, hypokalemia, and recurrent unexplained infections. Whole-exome sequencing identified a novel pathogenic nonsense variant in the *ARMC5* gene in this patient. Together with a systematic review of the published literature, this study discusses the clinical diagnosis, molecular pathogenesis, and management strategies of *ARMC5*-related PBMAH.

Accumulating evidence indicates that *ARMC5*-related PBMAH typically presents with mild or subclinical autonomous cortisol excess rather than overt Cushing syndrome (CS) (4). Most patients exhibit only mildly elevated 24-hour urinary free cortisol levels, partial resistance to the low-dose dexamethasone suppression test (DST), and disruption of the circadian cortisol rhythm, while lacking the classic features of CS, such as moon facies, abdominal purple striae, or muscle atrophy (4). Consequently, the clinical manifestations of PBMAH are highly insidious, and patients are often initially treated for long-standing metabolic complications rather than for the underlying adrenal disorder. It has been estimated that more than 70% of patients with PBMAH develop refractory hypertension, and more than 50% progress to type 2 diabetes mellitus as a consequence of long-term untreated cortisol excess (4).

The clinical presentation of this patient was highly consistent with the epidemiological characteristics described above: despite receiving standard pharmacological therapy, the patient had a 10-year history of poor glycemic control and hypertension, without overt CS-specific features, which likely contributed to the delayed diagnosis. Endocrine function testing showed suppressed plasma ACTH levels, loss of the circadian cortisol rhythm, and failure of suppression after a low-dose DST, all of which supported the diagnosis of ACTH-independent cortisol excess associated with PBMAH. Contrast-enhanced abdominal computed tomography (CT) revealed marked irregular bead-like enlargement of both adrenal glands, with multiple round low-attenuation lesions, the largest of which measured >10 mm in diameter, thereby fulfilling the imaging diagnostic criteria for PBMAH, namely diffuse enlargement of both adrenal glands with multiple nodules ≥1 cm (5). On the basis of endocrine function testing, imaging findings, and genetic sequencing, a final diagnosis of PBMAH was established.

Germline and somatic inactivating variants in the *ARMC5* gene are considered the most common genetic causes of PBMAH, reported in up to approximately 80% of familial cases and 20%–25% of sporadic cases (6, 7). Previous studies have identified various pathogenic *ARMC5* variants in patients with PBMAH (1, 2, 5, 6), including nonsense, frameshift, splice-site, deletion, and deleterious missense variants. Most of these variants are loss-of-function or predicted deleterious variants, further supporting the role of *ARMC5* as an important tumor suppressor gene involved in the development and progression of PBMAH. *ARMC5* pathogenic variants are highly heterogeneous, ranging from single-nucleotide variants to large structural variants, and may lead to partial or complete loss of function of the encoded protein, thereby causing adrenocortical hyperplasia. Nonsense variants are among the most common pathogenic *ARMC5* variants; these variants introduce a premature termination codon into the coding sequence, leading to premature termination of translation and the production of truncated, unstable proteins. Truncated ARMC5 proteins usually lack key functional domains, resulting in complete loss of tumor suppressor function (3, 6).

At the molecular level, ARMC5 protein is a protein containing armadillo (ARM) repeats, with multiple ARM repeats in the central region and a BTB domain at the C-terminus (8). Recent studies have shown that *ARMC5* can form an active E3 ubiquitin ligase complex with Cullin 3 (CUL3) and RING-box protein 1 (RBX1). Under physiological conditions, this complex targets RPB1, the largest subunit of RNA polymerase II (encoded by the POLR2A gene), for ubiquitination and degradation, thereby regulating protein turnover and global transcriptional activity (8). When both alleles of *ARMC5* are inactivated, RPB1 ubiquitination is markedly reduced, resulting in abnormal accumulation of RPB1 and increased stability of the RNA polymerase II complex, which in turn up-regulates the transcription of several downstream target genes.

These upregulated genes include several key genes involved in steroid biosynthesis, such as steroidogenic acute regulatory protein (StAR) and various cytochrome P450 enzymes, including CYP11B1 and CYP17A1 (8).

Specifically, StAR mediates the rate-limiting step in steroid biosynthesis by transporting cholesterol from the cytoplasm to the mitochondrial inner membrane. Therefore, StAR overexpression provides sufficient substrate for subsequent hormone synthesis (9). CYP11B1 catalyzes the conversion of 11-deoxycortisol to cortisol, and its overexpression can directly increase cortisol production. CYP17A1 is a key enzyme in the synthesis of cortisol and sex hormone precursors, and its overexpression promotes the synthesis of cortisol and androgen precursors (10).

In this case, a heterozygous nonsense variant in *ARMC5* (c.2125C > T) was identified by whole-exome sequencing (WES), and Gln at position 709 was replaced by a premature stop codon (*), resulting in premature termination of translation and the generation of a truncated, nonfunctional ARMC5 protein, consistent with the pathogenic mechanism of *ARMC5* inactivation described above.

Pathogenic variants were interpreted according to the standards and guidelines of the American College of Medical Genetics and Genomics (ACMG) (11). The population frequency of the *ARMC5* c.2125C > T variant identified in this study is extremely low, at only 1.24 cases per million individuals. The variant is not recorded in ClinVar, suggesting that it is rare and has not been previously reported.

According to ACMG criteria, this variant was classified as pathogenic based on the following evidence: (1) the nonsense variant introduced a premature termination codon, resulting in complete loss of *ARMC5* function; (2) it is extremely rare in the general population; and (3) the patient's clinical and imaging findings were highly consistent with *ARMC5*-related PBMAH. Although co-segregation analysis could not be performed because the patient's relatives declined genetic testing, the available evidence was sufficient to support the pathogenic classification of this variant. Compared with previously reported cases, the present case has several distinctive features. The nonsense *ARMC5* variant identified in this patient, c.2125C > T (p.Q709*), has not been documented in public genetic databases or in the published literature to date. This finding expands the mutational spectrum of *ARMC5*-associated primary bilateral macronodular adrenal hyperplasia (PBMAH). In addition, the patient presented with prominent cortisol excess-related metabolic abnormalities, including diabetes mellitus and hypertension, which improved markedly after unilateral adrenalectomy. This case may therefore provide clinically relevant insights for diabetes specialists, particularly regarding the evaluation of refractory metabolic disorders in patients with bilateral adrenal lesions.

Currently, adrenalectomy remains the mainstay of treatment for PBMAH, aiming to control cortisol excess and improve metabolic complications. The choice between unilateral and bilateral adrenalectomy should be individualized according to the patient's clinical condition, and glucocorticoid replacement therapy may be required after surgery, particularly after bilateral adrenalectomy (12). This patient underwent left adrenalectomy, after which blood glucose and blood pressure were well controlled with standard medical therapy, suggesting a favorable postoperative outcome.

This study has several limitations. First, because the patient's family members declined genetic testing, familial co-segregation analysis could not be performed to assess whether this variant co-segregated with the disease phenotype. Second, this study was limited by its single-case design; therefore, the long-term prognosis of this patient and the clinical significance of this novel variant require further validation through additional case accumulation and long-term follow-up.

In summary, this case report describes a sporadic case of PBMAH caused by a novel pathogenic nonsense variant in *ARMC5*. This case highlights that PBMAH-related subclinical CS should be considered in patients with persistently poor glycemic and blood pressure control despite standard treatment, particularly in those with hypokalemia and recurrent unexplained infections, even in the absence of typical CS manifestations. Timely endocrine evaluation, adrenal imaging, and genetic testing may facilitate the accurate diagnosis of this rare disease and help guide therapeutic decision-making and genetic counseling for the proband's family members.

## Data Availability

The datasets presented in this article are not readily available due to patient confidentiality restrictions. The de-identified data supporting the conclusions of this case report are included within the main text of the article. Requests for additional de-identified data may be directed to the corresponding author.
